# Accelerating 3-D GPU-based Motion Tracking for Ultrasound Strain Elastography Using Sum-Tables: Analysis and Initial Results

**DOI:** 10.3390/app9101991

**Published:** 2019-05-15

**Authors:** Bo Peng, Shasha Luo, Zhengqiu Xu, Jingfeng Jiang

**Affiliations:** 1 School of Computer Science, Southwest Petroleum University, Chengdu 610500, China; 2 Department of Biomedical Engineering, Michigan Technological University, Houghton, MI 49931, USA

**Keywords:** motion tracking, ultrasound elastography, graphics processing unit, correlation, sum-table, block-matching

## Abstract

Now, with the availability of 3-D ultrasound data, a lot of research efforts are being devoted to developing 3-D ultrasound strain elastography (USE) systems. Because 3-D motion tracking, a core component in any 3-D USE system, is computationally intensive, a lot of efforts are under way to accelerate 3-D motion tracking. In the literature, the concept of Sum-Table has been used in a serial computing environment to reduce the burden of computing signal correlation, which is the single most computationally intensive component in 3-D motion tracking. In this study, parallel programming using graphics processing units (GPU) is used in conjunction with the concept of Sum-Table to improve the computational efficiency of 3-D motion tracking. To our knowledge, sum-tables have not been used in a GPU environment for 3-D motion tracking. Our main objective here is to investigate the feasibility of using sum-table-based normalized correlation coefficient (ST-NCC) method for the above-mentioned GPU-accelerated 3-D USE. More specifically, two different implementations of ST-NCC methods proposed by Lewis et al. and Luo-Konofagou are compared against each other. During the performance comparison, the conventional method for calculating the normalized correlation coefficient (NCC) was used as the baseline. All three methods were implemented using compute unified device architecture (CUDA; Version 9.0, Nvidia Inc., CA, USA) and tested on a professional GeForce GTX TITAN X card (Nvidia Inc., CA, USA). Using 3-D ultrasound data acquired during a tissue-mimicking phantom experiment, both displacement tracking accuracy and computational efficiency were evaluated for the above-mentioned three different methods. Based on data investigated, we found that under the GPU platform, Lou-Konofaguo method can still improve the computational efficiency (17–46%), as compared to the classic NCC method implemented into the same GPU platform. However, the Lewis method does not improve the computational efficiency in some configuration or improves the computational efficiency at a lower rate (7–23%) under the GPU parallel computing environment. Comparable displacement tracking accuracy was obtained by both methods.

## 1. Introduction

Ultrasound strain elastography (USE) [1] can provide new information than that contained in B-Mode ultrasound images, which display only the amplitudes of envelope detected and decimated echo signals. USE has been successfully used for breast lesion differentiation [2–4] because it is capable of visualizing elevated tissue hardness. The strain/modulus nonlinearity has been also explored by others in order to better characterize breast lesions [5–7]. Furthermore, a number of studies in the literature has been devoted to understanding signal quality [8], image resolution [9,10] and contrast [11,12] in USE. In this study, we solely focus on ultrasound-based motion tracking, because it plays a critically important role in USE.

Among all motion tracking algorithms [13], the block-matching algorithm is one of the most used correlation-based methods and has been adopted by several prototype USE systems [14–21] due to its simplicity. Furthermore, the block-matching algorithm can also be easily extended to include motion regularization or multi-scale tracking to deal with large tissue deformation. In the framework of block matching algorithm, similarity or correlation evaluation influences both motion tracking accuracy and computational efficiency [22]. The commonly used similarity/correlation evaluation metrics include sum absolute error, sum square error and (SSD), entropy, and normalized cross-correlation (NCC). NCC has been proved to be one of the best similarity matching criteria in block matching methods [22] and therefore, has been widely used in various ultrasound-based motion tracking algorithms.

One drawback of using the NCC as a correlation metric is that calculating NCC following the standard protocol is computationally intensive, thereby limiting its use in many ultrasound techniques requiring real-time performance. Lewis proposed to use a pre-computed table in conjunction with a fast Fourier Transform (FFT) to calculate NCC values. His method is often referred as to Sum-Table (ST) method now [23]. Luo et al. further modified Lewis’ approach [22] by replacing the requirement of using FFT with another ST. Both methods [22,23] have demonstrated that they can achieve almost the same accuracy together with significantly faster calculation efficiency in a serial computing environment in which i.e., calculations are conducted by a central processing unit (CPU).

Several major vendors (e.g., General Electric, Siemens, Philips, Hitachi, Toshiba, Samsung Medison, etc.) have released their commercially-available USE packages. However, those clinical USE systems all operate in 2D. However, continued efforts [16,19,24–28] are under the way to expand USE into 3-D because 3-D tracking can significantly improve quality of strain elastograms [16,19].

Now FDA-approved 3-D automated breast ultrasound (ABUS) systems (e.g., GE InveniaTM; Siemens Acuson S2000 ABVS) have been released. It is our vision that with the availability of GPU-acceleration, bringing 3-D breast USE into the clinical workflow becomes possible. In the last decade, GPU-based parallel computing has been utilized to accelerate ultrasound-based motion tracking in several important applications including USE [21,29–31], shear wave elastography [32] and color Doppler imaging [33]. In particular, the work by Peng et al. [21] demonstrated that high quality strain data can be obtained for a reasonably large volume (e.g., 2.5 × 2.5 × 2.5 cm^3^) in 20 s. Consequently, investigating whether or not the concept of ST can be used to further improve 3-D motion tracking is a logical next step.

To this end, our primary goal is to investigate the feasibility of using ST in conjunction with GPUs to further accelerate 3-D motion tracking. Thus, two above-mentioned ST methods (i.e., Lewis’ and Luo’s methods) were analyzed and implemented for parallel (GPU) and serial (CPU) computing settings. The standard protocol of NCC calculation without the use of ST was used as the baseline in both computing settings for a systematic comparison.

## 2. Materials and Methods

### 2.1. A Description of GPU-Accelerated Block-Matching Algorithm in USE

**Figure 1:**
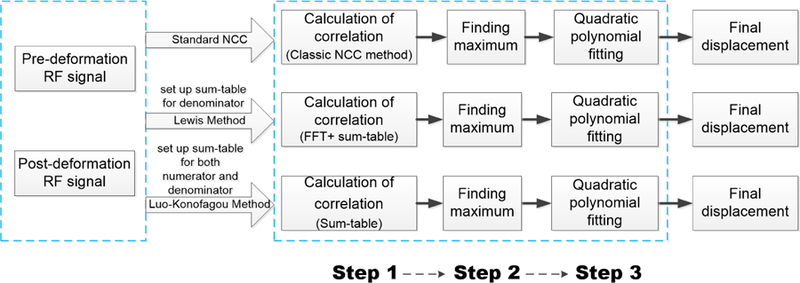
An illustrative diagram show how the calculation of NCC values with and without sum-tables are integrated into ultrasound-based motion tracking. RF signal stands for radiofrequency ultrasound echo signal.

As shown in Figure 1, in order to estimate one displacement vector (*dx*, *dy*, *dz*) for an arbitrary location (*x*, *y*, *z*) from a pair of pre- and post-deformation ultrasound echo volumes, the block-matching algorithm can be largely divided into three steps: (1) selecting a pair of reference and target ultrasound signals from two successive ultrasound radio frequency (RF) fields based on a predetermined search range and a tracking kernel; (2) calculating an NCC value between the pair of selected reference and target echo signals, generating a 3-D resultant NCC map for a given search range, and finding a peak from the NCC map; and (3) fitting NCC values around the peak NCC map to a 3-D quadratic surface [34] to find the estimated displacement vector. During the process, the tracking-kernel size and search range must be determined prior to the start of motion tracking. The location (*x*, *y*, *z*) here is referred to a location in the pre-deformation ultrasound each volume (i.e., the reference volume). Thus, the (*dx*, *dy*, *dz*) represents the displacement vector by which the medium moved to the post-deformation ultrasound echo volume (i.e., the target volume) from the reference volume. In the block-matching algorithm, time-domain ultrasound echo signals are used to calculate correlation.

As can be seen from the schematic diagram in Figure 1, Lewis’ and Luo’s methods both need to create sum tables prior to the calculation of NCC values in Step 1. Those sum-tables are used as “lookup-tables” to replace the standard NCC calculations in order to reduce the computing time. The details of Step 1 for three different ways of calculating NCC values are discussed below. Steps 2 and 3 are exactly the same for all three methods. Specifically, Step 2 is to find the maximum on the NCC map. Thus, an integer displacement vector can be determined for the location (*x*, *y*, *z*). Finally, in Step 3, the subs-sample displacement vector can be obtained by fitting a 3 × 3 × 3 matrix containing NCC values surrounding the maximum NCC value into a quadratic surface. Through Steps 2 and 3, the final axial, lateral and elevational displacements with sub-sampling precision can be obtained.

#### 2.1.1. A Standard Protocol for Calculating NCC

Given one reference signal *f* and one target signal *g*, the NCC function over a search range (*τ*_*x*_, *τ*_*y*_, *τ*_*z*_) can be calculated as follows:

(1) $$R_{NCC}\left(u,v,w,\tau_{x},\tau_{y},\tau_{z}\right)\;=\frac{\sum_{m=u}^{u+W_{x}-1}\sum_{n=v}^{v+W_{y}-1}\sum_{k=w}^{w+W_{z}-1}\left\{f\left(m,n,k\right)•g\left(m-\tau_{x},n-\tau_{y},k-\tau_{z}\right)\right\}}{\sqrt{\sum_{m=u}^{u+W_{x}-1}\sum_{n=v}^{v+W_{y}-1}\sum_{k=w}^{w+W_{z}-1}\left\{f^{2}\left(m,n,k\right)\right\}•\sum_{m=u}^{u+W_{x}-1}\sum_{n=v}^{v+W_{y}-1}\sum_{k=w}^{w+W_{z}-1}\left\{g^{2}\left(m-\tau_{x},n-\tau_{y},k-\tau_{z}\right)\right\}}}$$

where the dimensions of the reference and target windows (tracking kernels) are [*u*, *u* + *W*_*x*_ *−* 1], [*v*, *v* + *W*_*y*_ *−* 1], [*w*, *w* + *W*_*z*_ *−* 1] in the lateral, axial and elevational directions, respectively. Similarly, (*W*_*x*_, *W*_*y*_, *W*_*z*_) are the tracking kernel dimensions in lateral, axial and elevational directions, respectively. In Equation (1), the origin of the reference window (tracking kernel) is (*u*, *v*, *w*) and [*τ*_*x*_, *τ*_*y*_, *τ*_*z*_] is the search range defined below,

(2) $$\left(\tau_{1}\leq \tau_{x}\leq \tau_{2},\tau_{3}\leq \tau_{y}\leq \tau_{4},\tau_{5}\leq \tau_{z}\leq \tau_{6}\right)$$

In the block-matching algorithm, [*τ*_1_, *τ*_2_], [*τ*_3_, *τ*_4_] and [*τ*_5_, *τ*_6_] are pre-determined by the algorithm. For each 3-D shift (*τ*_*x*_, *τ*_*y*_, *τ*_*z*_), Equation (1) can be used to obtain one NCC value. Thus, looping through the entire search range yields a 3-D NCC map.

#### 2.1.2. Lewis’ Sum-Table Method

When the block matching algorithm is used, significant overlaps among adjacent tracking kernels exist, resulting in a lot of redundant computation. Thus, Lewis proposed to use pre-computed tables (also known as Sum-tables) to partially “memorize” some correlation between *f* and *g* [23]. Together with Fourier Transform (FT), Lewis’ method can conceptually reduce the computational demands. More specifically, the numerator in Equation (1) is a convolution of the tracking kernel within the reference signal *f* with the corresponding tracking kernel of the reversed target signal *g*(*−m*, *−n*, *−k*) and can be computed by Fourier Transform (FT). The process is defined as follows:

(3) $$\sum_{m,n,k}f\left(m,n,k\right)•g\left(m-\tau_{x},n-\tau_{y},k-\tau_{z}\right)=F^{-1}\left\{F\left(f\right)F^{*}\left(g\right)\right\}$$

where F stands for an FT operator. The complex conjugate accomplishes reversal of the template via the FT’s time reversal property [23]. Equation (3) was implemented via fast Fourier Transform (FFT) and thus, *f* and *g* were zero-padded to a common power of two.

Now referring to the calculation of the denominator in Equation (1), two sum tables were used: one for the reference signal *f* and the other for the target signal *g*. Let $s_{f}^{2}\left(u,v,w\right)$ and $s_{g}^{2}\left(u,v,w\right)$ denotes the created sum-tables for *f* and *g*, respectively. More details about setting up these two sum-table can be found in Appendix A. Once these two tables become available, the calculation of denominator can be conducted through a very efficient manner as follows,

(4) $$\sum_{m=u}^{u+W_{x}-1}\sum_{n=v}^{v+W_{y}-1}\sum_{k=w}^{w+W_{z}-1}f^{2}\left(m,n,k\right)\;=s_{f}^{2}\left(u+W_{x}-1,v+W_{y}-1,w+W_{z}-1\right)\;-s_{f}^{2}\left(u+W_{x}-1,v+W_{y}-1,w-1\right)\;-s_{f}^{2}\left(u+W_{x}-1,v-1,w+W_{z}-1\right)\;-s_{f}^{2}\left(u-1,v+W_{y}-1,w+W_{z}-1\right)\;+s_{f}^{2}\left(u-1,v-1,w+W_{z}-1\right)\;+s_{f}^{2}\left(u-1,v+W_{y}-1,w-1\right)\;+s_{f}^{2}\left(u+W_{x}-1,v-1,w-1\right)\;-s_{f}^{2}\left(u-1,v-1,w-1\right)$$

(5) $$\sum_{m=u}^{u+W_{x}-1}\sum_{n=v}^{v+W_{y}-1}\sum_{k=w}^{w+W_{z}-1}g^{2}\left(m+\tau_{x},n+\tau_{y},k+\tau_{z}\right)\;=s_{g}^{2}\left(u+W_{x}-1+\tau_{x},v+W_{y}-1+\tau_{y},w+W_{z}-1+\tau_{z}\right)\;-s_{g}^{2}\left(u+W_{x}-1+\tau_{x},v+W_{y}-1+\tau_{y},w-1+\tau_{z}\right)\;-s_{g}^{2}\left(u+W_{x}-1+\tau_{x},v-1+\tau_{y},w+W_{z}-1+\tau_{z}\right)\;-s_{g}^{2}\left(u-1+\tau_{x},v+W_{y}-1+\tau_{y},w+W_{z}-1+\tau_{z}\right)\;+s_{g}^{2}\left(u-1+\tau_{x},v-1+\tau_{y},w+W_{z}-1+\tau_{z}\right)\;+s_{g}^{2}\left(u-1+\tau_{x},v+W_{y}-1+\tau_{y},w-1+\tau_{z}\right)\;+s_{g}^{2}\left(u+W_{x}-1+\tau_{x},v-1+\tau_{y},w-1+\tau_{z}\right)\;-s_{g}^{2}\left(u-1+\tau_{x},v-1+\tau_{y},w-1+\tau_{z}\right)$$

#### 2.1.3. Luo-Konofagou Sum-Table Method

In Luo and Konofagou method [35], both the numerator and denominator of Equation (1) are calculated using sum-tables. In addition to Equations (4) and (5), the numerator can be calculated as follow using a set of sum-tables *s*_*f*, *g*_(*u*, *v*, *w*) to look up pre-computed numbers,

(6) $$\sum_{m=u}^{u+W_{x}-1}\sum_{n=v}^{v+W_{y}-1}\sum_{k=w}^{w+W_{z}-1}f\left(m,n,k\right)•g\left(m+\tau_{x},n+\tau_{y},k+\tau_{z}\right)\;={\text{s}}_{f,g}\left(u+W_{x}-1,v+W_{y}-1,w-1+W_{z},\tau_{x},\tau_{y},\tau_{z}\right)\;-{\text{s}}_{f,g}\left(u+W_{x}-1,v+W_{y}-1,w-1,\tau_{x},\tau_{y},\tau_{z}\right)\;-{\text{s}}_{f,g}\left(u+W_{x}-1,v-1,w-1+W_{z},\tau_{x},\tau_{y},\tau_{z}\right)\;-{\text{s}}_{f,g}\left(u-1,v+W_{y}-1,w-1+W_{z},\tau_{x},\tau_{y},\tau_{z}\right)\;+{\text{s}}_{f,g}\left(u-1,v-1,w-1+W_{z},\tau_{x},\tau_{y},\tau_{z}\right)\;+{\text{s}}_{f,g}\left(u-1,v+W_{y}-1,w-1,\tau_{x},\tau_{y},\tau_{z}\right)\;+{\text{s}}_{f,g}\left(u+W_{x}-1,v-1,w-1,\tau_{x},\tau_{y},\tau_{z}\right)\;-{\text{s}}_{f,g}\left(u-1,v-1,w-1,\tau_{x},\tau_{y},\tau_{z}\right)$$

According to Equations (4)–(6), the calculation of NCC can be simplified to addition and subtraction operations. More formal analyses of algorithmic complexity and memory requirements can be found in Appendices B and C.

### 2.2. GPU Implementation of Three NCC Calculation Methods

#### 2.2.1. A Brief Description of GPU Computing

CUDA (Compute Unified Device Architecture) is a common parallel computing architecture launched by Nvidia (Nvidia Inc., Santa Clara, CA, USA). This architecture enables GPUs to solve complex computing problems in parallel. In CUDA, a KERNEL function is defined as a function that performs multi-threaded parallel computation. Similarly, a DEVICE function is defined as a single-threaded function called by a KERNEL function on GPU. According to the memory structure defined in CUDA, off-chip memory (global memory and texture memory) has a higher access delay than on-chip memory (register, shared memory and constant memory). Consequently, the on-chip memory should be given priority during programming in order to improve memory access efficiency. It is worth noting that the lower-case “kernel” is used for tracking kernels in this study, while the upper-case “KERNEL” is referred as to a GPU KERNEL function.

#### 2.2.2. Block-Matching Using GPU Parallel Computing

The block-matching algorithm implemented in this study is the classic block-matching algorithm. Because of memory limitation, full 3-D block-matching tracking was first performed in a slice-by-slice manner. Thus, a full displacement vector field for a single slice consists of *M × N* (Axial × Lateral) displacement estimation locations; each displacement estimation location obtains one 3-D displacement vector after the block-matching process. Because tracking displacements using the classic block-matching algorithm for each location is independent (i.e., no data dependency and requirements regarding communication), in the CUDA programming structure, the *M × N* thread can be launched through the KERNEL function (see Section 2.2.1). As shown in Figure 1, in order to construct 3-D NCC map for each displacement estimation location, one NCC value needs to be calculated at a specified search location (*τ*_*x*_, *τ*_*y*_, *τ*_*z*_). In the **Step 1** (see Figure 1), given a search range (*A*[*Axial*], *B*[*Lateral*], *C*[*Elevational*]), the KERNEL function can start (*M × N*) × (*A × B × C*) CUDA threads to obtain one 3-D NCC map to complete Step 1. Here, *A* = (*τ*_2_ *− τ*_1_ + 1), *B* = (*τ*_4_ *− τ*_3_ + 1) and *C* = (*τ*_6_ *− τ*_5_ + 1).

In the subsequent **Steps 2** and **3** (see Figure 1), the number of CUDA threads in the KERNEL functions are consistent with the total number of displacement estimation locations *M × N*. Basically, each CUDA thread invokes a DEVICE function to search the peak NCC value of the corresponding 3-D NCC map (Step 2). Then, the same DEVICE function uses a quadratic fitting to obtain sub-sample estimates (Step 3) [36].

In the process of implementation, a few notable strategies were used. First, one-dimensional thread structure was adopted to ensure the consistency of memory access. In other words, we attempted to ensure that adjacent threads read adjacent memory regions and try to satisfy the requirement of coalesced memory access. Second, RF data were stored in TEXTURE memory. Nvidia GPU TEXTURE memory technology can avoid the delay caused by cross-line reading. Third, some important variables (such as axial and lateral search range, tracking kernel size) are stored in GPU constant memory for rapid accesses.

#### 2.2.3. Implementing A Standard NCC Calculation on CUDA

Under the above-mentioned parallel implementation framework, improving the efficiency for memory access was the most important consideration. This is because the calculation of a 3-D NCC map will read each RF sample multiple times. In order to avoid this frequent data reading from TEXTURE memory, we load each small part of RF echo data into on-chip memory during CUDA programming to improve memory access efficiency. The detailed GPU implementation of this method is described in our early work [21].

#### 2.2.4. Implementing Lewis’ Method on CUDA

In this study, the FFT function library cuFFT available in CUDA (Nvidia Inc., CA, USA) was used to calculate the numerator of Equation (1). The cuFFT library provides a simple interface for computing FFTs on Nvidia GPUs. Also, the cuFFT library has been highly optimized and systematically tested for the GPU parallel computing environment. In contrast, FFTW (http://www.fftw.org/) is a state-of-art fast FFT toolbox on CPU and was applied to implement Lewis’s method on CPU.

The calculation of the denominator in Equation (1) needs two sum-tables: one for the reference RF signal and the other one for the target RF signal. In this study, a parallel scan algorithm proposed by Sengupta et al. [37] was adopted to perform rapid prefix sum (i.e., cumulative sum) computation for each direction. The scan algorithm is based on an idea of balanced tree proposed by Blelloch [38]. Equations can be found in Appendix A.

**Figure 2:**
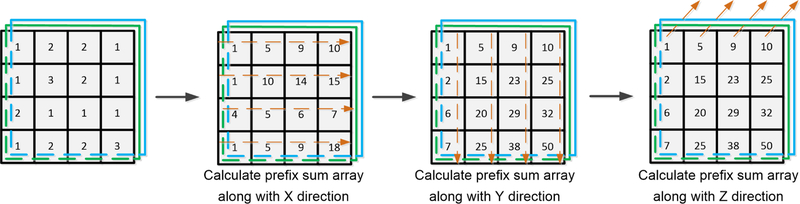
An illustration of calculating the 3-D sum-table under the GPU parallel computing environment.

More specifically, the prefix sum builds a balanced binary tree on the input data and scans it from the top to the root for calculating the prefix sum. Figure 2 illustrates the process of setting up a 3-D sum-table. The calculation of a 3-D sum-table can be divided into the following three stages: (1) constructing a prefix sum array along with the X-axis direction; (2) constructing the prefix sum array along with the Y-axis direction based on the result of (1); and (3) using the prefix scan algorithm to build the prefix sum array along with the Z-axis direction. A for-loop operation involving the above-mentioned three stages is sufficiently fast to establish the final 3-D sum-table. Consequently, the prefix scan algorithm was only invoked once for each sum-table and twice during the entire process. If the size of the 3-D RF signal is (*Rows × Columns × Slices*), the corresponding KERNEL function will launch (*Rows × Columns × Slices*) CUDA threads in order to create a sum-table.

After setting up 3-D sum-tables, the calculations of numerator and denominator (see Equation (1)) are accomplished by FFT and through two sum-tables, respectively.

#### 2.2.5. Implementing Luo-Konofagou Method

Please recall that in the Luo-Konofagou method, computing the denominator in Equation (1) is the same as Lewis’s method (see Section 2.2.4). Luo-Konofagou method replaced the FFT operation to sum-table method for computing the numerator. During this process, numerator’s calculation needs (*τ*_2_ *− τ*_1_ + 1) × (*τ*_4_ *− τ*_3_ + 1) × (*τ*_6_ *− τ*_5_ + 1) (i.e., *A × B × C*) sum-tables to represent the standard cross-correlation (CC) between the reference and target RF signals at a specified search location (*τ*_*x*_, *τ*_*y*_, *τ*_*z*_). Thus, we need to launch (*τ*_2_ *− τ*_1_ + 1) × (*τ*_4_ *− τ*_3_ + 1) × (*τ*_6_ *− τ*_5_ + 1) × (*Rows × Columns × Slices*) CUDA threads. It is easy to see that the number of threads and required memory become a burden to manage if the search range is very large.

### 2.3. Experimental Design and Data Analysis

#### 2.3.1. A Tissue-Mimicking Phantom Experiment

**Figure 3:**
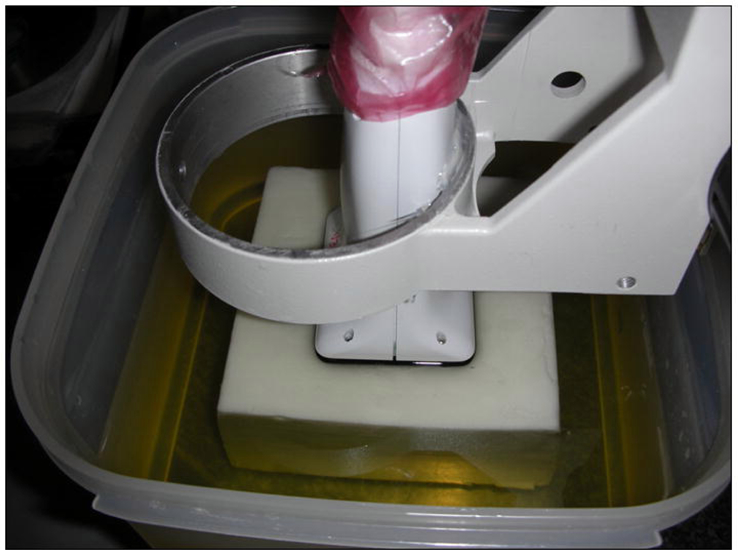
A photograph showing the CMUT ultrasound transducer and the tissue-mimicking phantom. The transducer is attached to a fixture that can be moved in the axial direction in order for the transducer to compress the phantom. The original picture was published in [19] and reuse permission has been granted for work presented in this paper.

Volumetric ultrasound data acquired from a 100 mm × 100 mm × 70 mm oil-in-gelatin phantom were also used to test the above-mentioned three different NCC calculation methods. The inclusion in the ultrasound data was 5 times stiffer than the background. As shown in Figure 3, a 9-MHz CMUT transducer connected to a Siemens Antares (Siemens Health Care, Inc. Ultrasound Division, Mountain View, CA, USA) was used to acquire radio frequency (RF) echo data. A robotic arm (see Figure 3) was used to move the CMUT transducer downward to compress the phantom. The volume-to-volume deformation was approximately 1.5%. Then, ultrasound echo data before and after the compression were first acquired using ultrasound research interface (URI, Siemens Health Care, Inc. Ultrasound Division, Mountain View, CA, USA) installed on the Siemens scanner. Each echo data represented a 40 mm (axial) × 37 mm (lateral) × 30 mm (elevation) volume. The RF sample size, line spacing and distance between two parallel image planes were 0.0193-mm, 0.119-mm and 0.214-mm, respectively. More details can be found elsewhere [19].

#### 2.3.2. Data Analysis

The GPU hardware used in this study is Nvidia GeForce GTX TITAN X (Nvidia Corp., Santa Clara, CA, USA). The GPU card comes along with 80 Stream Multiprocessors, along with a total of 5120 CUDA computing cores along with 12 GB of Memory. The GPU card is installed on a desktop workstation with a Ubuntu operating system (version 16.04), Intel(R) Core(TM) i7–8700 CPU @ 3.20 GHz, and 16 GB of host memory. ANSI C and CUDA 9.0 were adopted for implementing all CPU and GPU algorithms. All testing was done under the MATLAB platform (Version 2016b, Mathworks Inc., Natick, MA, USA) and all implemented algorithms were invoked in the MATLAB environment through the MEX interface.

In total, six different implementations were done in this study: standard NCC in CPU, standard NCC in GPU, Lewis’ method in CPU and Lewis’ method in GPU, Luo-Konofagou method in CPU and Luo-konofagou method in GPU. Hereafter, those six implementations are referred to as **NCC-CPU**, **Lou-Konofagou-CPU**, **Lewis-CPU**, **NCC-GPU**, **Luo-Konofagou-GPU**, and **Lewis-GPU**, respectively. In this paper, the performance is mainly compared from the following two aspects: (1) Evaluating whether or not a different implementation yields substantial errors and (2) comparing computational efficiency of those three methods, given motion tracking parameters and the computing environment (i.e., CPU or GPU).

## 3. Results

### 3.1. Comparisons of Accuracy Among Different Implementations

**Figure 4:**
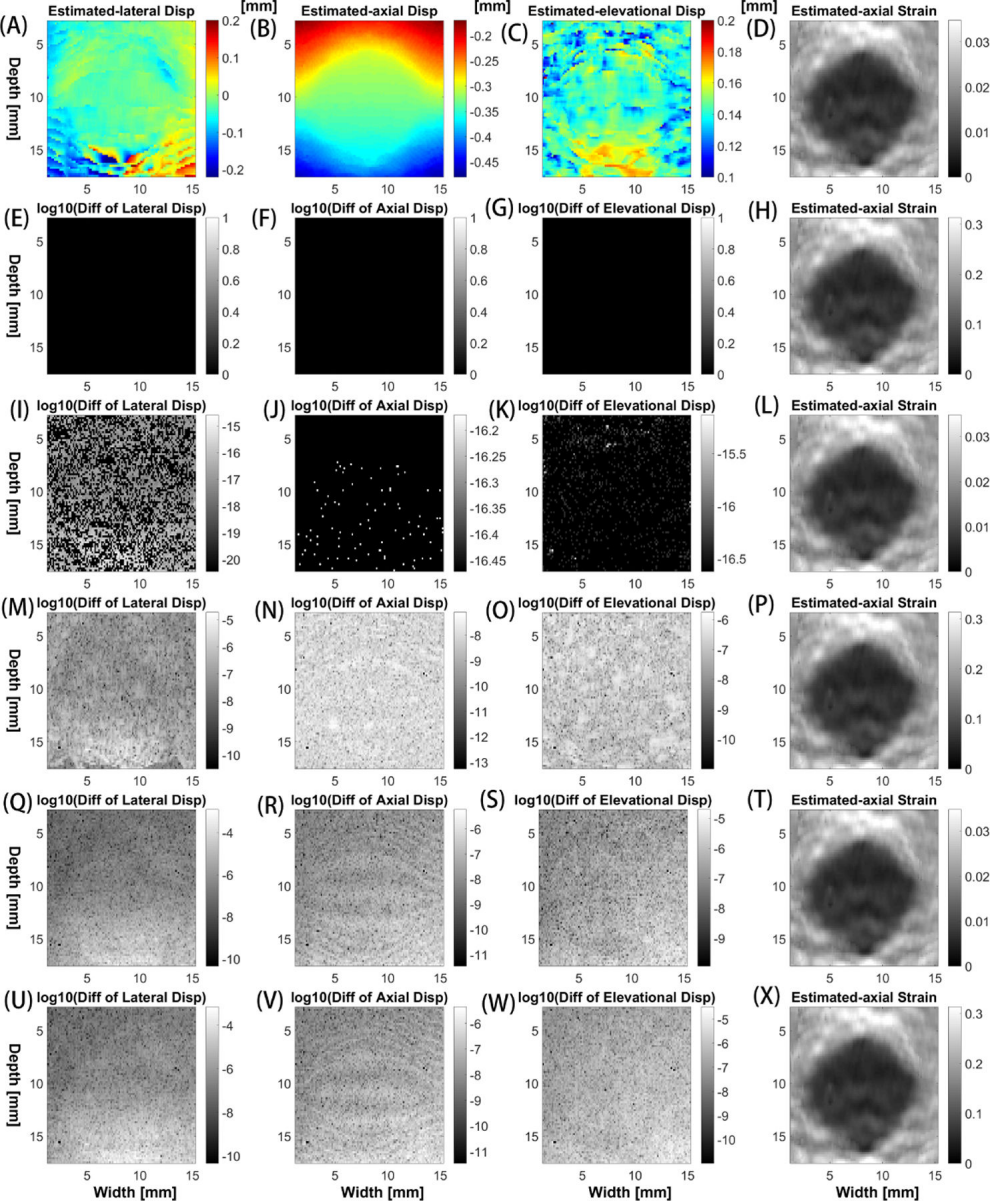
In the first row, (**A**) lateral, (**B**) axial and (**C**) elevational displacements estimated from a TM phantom using the CPU-Version of the standard NCC method. The (displacement) difference images from the first three columns of the second to the sixth rows showing the displacement difference between the stand-NCC-CPU method and one of the other five methods: [second row] Lou-Konofagou-CPU, [third row] Lewis-CPU, [fourth row] NCC-GPU, [fifth row] Lou-Konofagou-GPU and [sixth row] Lewis-GPU. Estimated displacement differences are displayed in a compressed (log_10_) fashion except for the results in the second row in which the differences are zeros. Disp. is an abbreviation of displacement. Axial strain images (**D**, **H**, **L**, **P**, **T**, and **X**) obtained by six implementations are shown in the fourth column.

**Figure 5:**
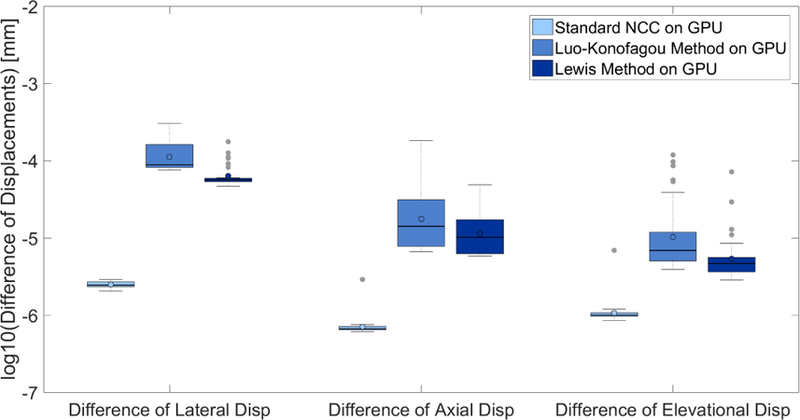
Clustered box plots illustrating absolute displacement difference values obtained from the standard-NCC-CPU implementation and three different GPU implementations (standard-NCC-GPU, Lou-Konofagou-GPU, Lewis-GPU). The top and bottom of the boxes indicate 75 and 25 percentiles, respectively. The line through the middle of each box represents the median. The error bars show the minimum and maximum values. The gray “dot-shaped” markers indicate outliers.

Displacement estimates obtained from using the **NCC-CPU** of the block-matching algorithm were compared to other five above-mentioned implementations: **Lou-konofagou-CPU**, **Lewis-CPU**, **NCC-GPU**, **Lou-Konofagou-GPU**, **Lewis-GPU**. In Figure 4, the standard-NCC-CPU implementation yielded exactly same displacements as compared to the Lou-Konofagou-CPU implementation (see the second row). The differences between the standard-NCC-CPU and Lewis-CPU implementations were neglectable (<10^*−*15^ mm), as shown in the third row of Figure 4. However, when comparing three GPU implementations (NCC-GPU, Lou-Konofagou-GPU and Lewis-GPU) to the standard-NCC-CPU implementation, small differences exist (see the fourth to sixth rows in Figure 4). The differences are quantitatively analyzed from 30 (elevational) slices of ultrasound displacement data and the result is shown in Figure 5.

It is clear from Figure 5 that all three GPU implementations produced slightly different displacement results. However, the difference was small (10^*−*6^–10^*−*4^ mm). Such a small difference did not result in visible differences on respective axial strain images (see the fourth column in Figure 4).

#### Computational Efficiency

**Figure 6:**
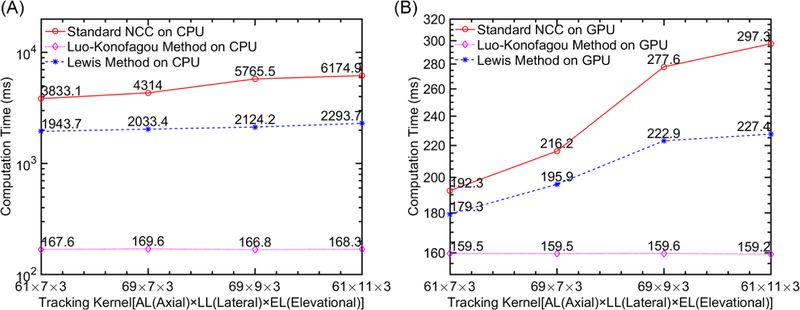
Plots comparing computational efficiency under two different environments: (**A**) CPU and (**B**) GPU. In (**A**), the computing time is displayed in log_10_ scale. Computing time was estimated based on the completion of 3-D motion tracking for a single slice of ultrasound data.

The influence of the tracking kernel size was investigated for six implementations and the results are shown in Figure 6. The search range was set to (5 [lateral] × 18 [axial] × 3 [elevational]) for tracking approximately 1.5% tissue deformation, and the size of tracking kernel varied as stated in those figures. For reference, 18, 61 and 69 axial samples are equivalent to 0.36 mm, 1.16 mm, and 1.35 mm, respectively, while 7, 9 and 11 beamlines are 0.83 mm, 1.07 mm and 1.31 mm, respectively. Three elevation planes equal to 0.64 mm in space. We found that the Lou-Konofagou method can substantially reduce the computing time under the CPU environment (i.e., >95% reduction between Lou-Konofagou-CPU and NCC-CPU). However, under the GPU environment, the reductions become small but still noticeable (between 17–46%). It is interesting to note that, under the GPU environment, Lewis’s method reduces the computing time at a lower rate (between 7–23%), as compared to Lou-Konofagou method.

**Figure 7:**
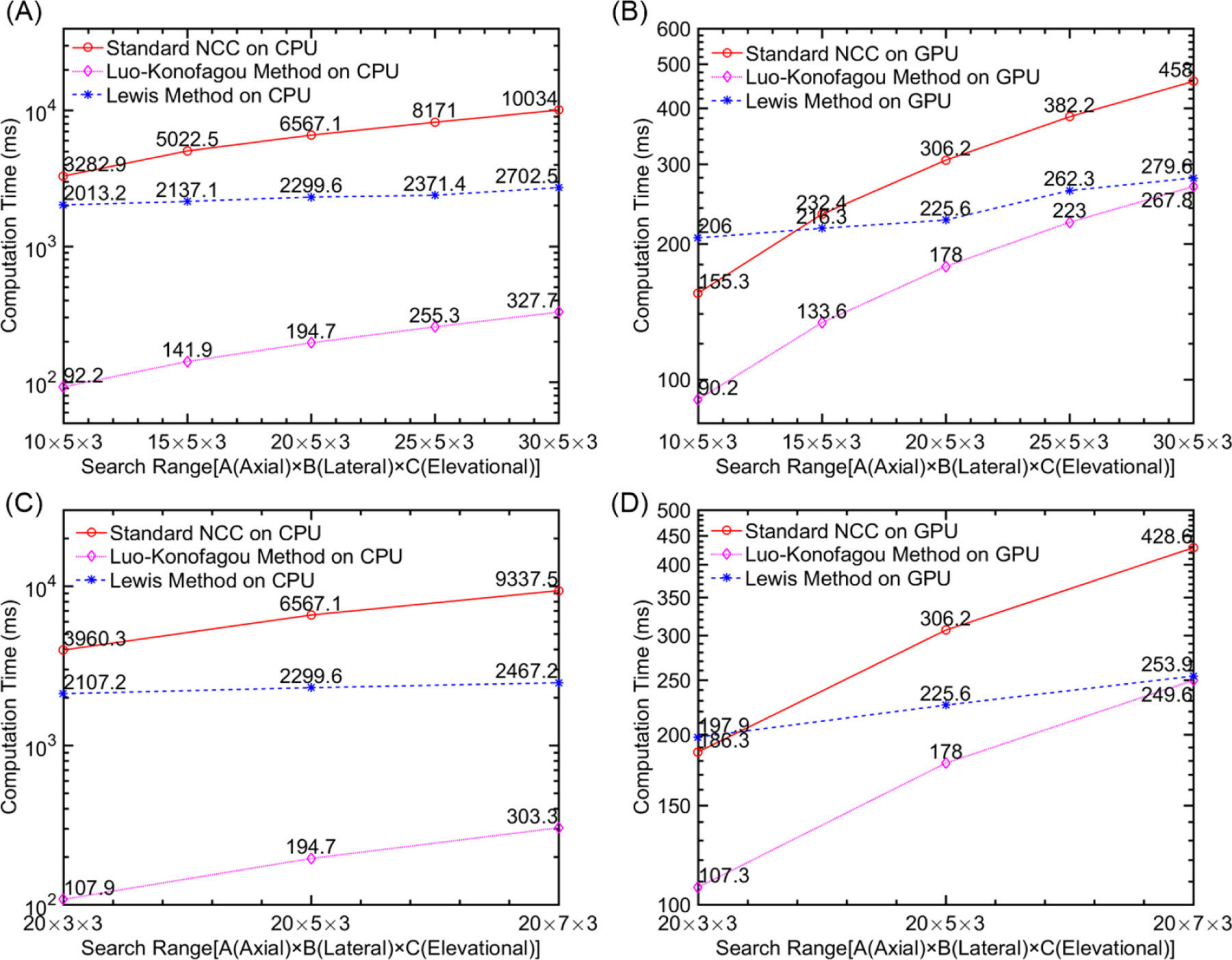
Plot showing computing time when the search range changed under the (**A**,**C**) CPU and (**B**,**D**) GPU environments. In all plots, the computing time is displayed in log_10_ scale. Computing time was estimated based on the completion of 3-D motion tracking for a single slice of ultrasound data.

When the tracking kernel was fixed at 9 [1.07 mm; lateral] × 69 [1.35 mm; axial] × 3 [0.64 mm; elevational]). The computational efficiency was also examined when the search range had been varied. It is common to change the search range in USE to accommodate different frame-to-frame (2D) or volume-to-volume (3-D) strain levels occurring in vivo. As shown in Figure 7, the required time to complete the motion tracking as the increase of search range. However, the trend of time reduction between the standard NCC method and the Lou-Konofagou method remained the same. In contrast, with the increase of the search range, time needed for Lewis’ method to complete the required tracking remains relatively steady (Figure 7).

As shown in Table 1, the standard deviation values among 30 slices were low as compared to the mean value. This indicated that the time required to calculate NCC values was stable regardless of the implementation used.

## 4. Discussion and Summary

In this paper, the motion tracking accuracy and computational efficiency of the three methods in the CPU serial computing environment and GPU parallel computing environment are compared and systematically analyzed. Under the CPU environment, the Lou-Konofagou method can substantially improve computational efficiency (by 95% or more). In contrast, using Luo-Konofagou method, the 3-D tracking can still be accelerated under the GPU parallel environment. However, the rate of improvements only ranged between 17% and 46% (see Figure 6). The rate of improvements obtained by the Lewis’ method was considerably less (7–23%; see Figure 6).

In order to further improve the GPU-based 3-D motion tracking, one strategy is to enhance the memory access efficiency. That requires us to make full use of on-chip memory. Recall that, according to Nvidia’s GPU specifications, the latency of on-chip memory is significantly better than that of off-chip memory. However, on-chip memory capacity is limited on GPUs. With the development of GPU hardware technology, the on-chip memory capacity may continue to increase. At the same time, the current GPU implementation can also be further optimized. In multi-instruction and multi-data (MIMD) mode, when one instruction is waiting for loading data, another segment of data can be used to perform some computing tasks at the same time. Therefore, the MIMD mode may further improve the computational efficiency of GPU implementation. We expect that with the improvements of GPU hardware, real-time 3-D ultrasound motion tracking may become a reality in the near future.

As the tissue deformation increases, the required search range becomes inevitably large. Consequently, the creation of those sum-tables requires a longer time (see Appendix B). It is also found that the Lou-Konofagou method demands a high usage of memory (see Appendix C). Also, when the search range increases, the number of required sum-tables becomes bigger accordingly. In this sense, in order to deal with large tissue deformation, the Luo-Konofagou method can be used in conjunction with a multiple-compression tracking strategy [39,40]. This is because the multi-compression method can effectively reduce the required search range at the expense of performing motion tracking multiple times. Alternatively, the Luo-Konofagou method can be used together with a 3-D region-growing motion tracking method [20]. The advantage of using a region-growing motion tracking method is that the search region would be fairly small. Both directions will be explored in our future work.

Our preliminary results demonstrated that the Lewis method accelerated the 3-D motion tracking at a slow rate (see Figures 6 and 7) in both CPU and GPU computing environments. This is largely because the FFT requires a large number of calculations in order to estimate the numerator in Equation (1) regardless of the computing environment.

## Figures and Tables

**Table 1.** A summary of computing time (mean *±* one standard deviation) using 6 different implementations. 3D Displacement fields were first calculated for 30 slices. Then, the mean values (*±* one standard deviation) were derived and displayed below. The tracking kernel size and search region were 69 × 9 × 3 and 18 × 5 × 3, respectively.

Implementation Method	Computing Time (milliseconds)
Standard-NCC-CPU	5760.0 ± 9.3
Luo-Konofagou-CPU	168.5 ± 2.2
Lewis-CPU	2120.0 ± 5.5
Standard-NCC-GPU	278.1 ± 0.9
Luo-Konofagou-GPU	159.9 ± 0.4
Lewis-GPU	225.2 ± 3.2

## Appendix A. Setting up 3-D Sum-Tables for 3-D Ultrasound Echo Data

The sum tables in this study are defined as follows:

(A1) $$s_{f}^{2}=\sum_{m=0}^{u}\sum_{n=0}^{v}\sum_{k=0}^{w}f^{2}\left(m,n,k\right)\;s_{g}^{2}=\sum_{m=0}^{u}\sum_{n=0}^{v}\sum_{k=0}^{w}g^{2}\left(m,n,k\right)\;s_{f,g}\left(u,v,w,\tau_{x},\tau_{y},\tau_{z}\right)=\sum_{m=0}^{u}\sum_{n=0}^{v}\sum_{k=0}^{w}f\left(m,n,k\right)\cdot g\left(m+\tau_{x},n+\tau_{y},k+\tau_{z}\right)$$

Above definitions are similar to those defined by Luo and Konofaguo in 2D [22]. The 3-D sum tables can be constructed efficiently using the following three steps. First, the summation is performed in the *u* (lateral) direction for each *v* and *w*. Then, the summation was performed in the *v* (axial) direction for each *u* and *w*. Finally, the summation was performed in the *w* (elevational) direction for each *u* an2d *v*. One illustrative example is shown in Figure 2. The constructing process of $s_{f}^{2}$, $s_{g}^{2}$ and $s_{f,g}^{2}$ are given by (A1)–(A3) below.

(A2) $$s_{f,temp\_u}^{2}\left(u,v,w\right)=f_{u,v,w}^{2}+s_{f,temp\_u}^{2}\left(u-1,v,w\right)\;s_{f,temp\_v}^{2}\left(u,v,w\right)=s_{f,temp\_u}^{2}\left(u,v,w\right)+s_{g,temp\_v}^{2}\left(u,v-1,w\right)\;s_{f}^{2}\left(u,v,w\right)=s_{f,temp\_v}^{2}\left(u,v,w\right)+s_{f}^{2}\left(u,v,w-1\right)$$

(A3) $$s_{g,temp\_u}^{2}\left(u,v,w\right)=g_{u,v,w}^{2}+s_{g,temp\_u}^{2}\left(u-1,v,w\right)\;s_{g,temp\_v}^{2}\left(u,v,w\right)=s_{g,temp\_u}^{2}\left(u,v,w\right)+s_{g,temp\_v}^{2}\left(u,v-1,w\right)\;s_{g}^{2}\left(u,v,w\right)=s_{g,temp\_v}^{2}\left(u,v,w\right)+s_{g}^{2}\left(u,v,w-1\right)$$

(A4) $$s_{f,g,temp\_u}\left(u,v,w,\tau_{x},\tau_{y},\tau_{z}\right)=f\left(u,v,w\right)\cdot g\left(u+\tau_{x},v+\tau_{y},w+\tau_{z}\right)+s_{f,g,temp\_u}\left(u-1,v,w,\tau_{x},\tau_{y},\tau_{z}\right)\;s_{f,g,temp\_v}\left(u,v,w,\tau_{x},\tau_{y},\tau_{z}\right)=s_{f,g,temp\_u}\left(u,v,w,\tau_{x},\tau_{y},\tau_{z}\right)+s_{f,g,temp\_v}\left(u,v-1,w,\tau_{x},\tau_{y},\tau_{z}\right)\;s_{f,g}\left(u,v,w,\tau_{x},\tau_{y},\tau_{z}\right)=s_{f,g,temp\_v}\left(u,v,w,\tau_{x},\tau_{y},\tau_{z}\right)+s_{f,g}\left(u,v,w-1,\tau_{x},\tau_{y},\tau_{z}\right)$$

## Appendix B. Analysis of Algorithmic Complexity for Sum-Table Methods

The complexity of calculating one NCC for 3-D block-matching is analyzed in the appendix for the sake of completeness. The analysis of complexity for the 3-D case is an extension to the work done by Briechle and Hanebeck in 2D [41]. Under the framework of a 3-D block-matching, one-direction search ranges are *S*_*x*_, *S*_*y*_ and *S*_*z*_ for lateral, axial and elevation directions, respectively. Of note, the one-direction search range above was defined as the maximal translation of the center of the tracking kernel along one given direction (e.g., upward or downward). Given a 3-D tracking kernel with a size of *W*_*x*_ [lateral], *W*_*y*_ [axial] and *W*_*z*_ [elevational] samples, we need to get access to a segment of RF data whose size is at least *M*_*x*_[*lateral*] *× M*_*y*_[*axial*] *× M*_*z*_[*elevational*] RF samples in order to complete the calculations for one NCC values (see Equation (1)), where *M*_*x*_ = 2*S*_*x*_ + *W*_*x*_ + 1, *M*_*y*_ = 2*S*_*y*_ + *W*_*y*_ + 1 and *M*_*z*_ = 2*S*_*z*_ + *W*_*z*_ + 1.

According to the given data, Table A1 shows the complexity for computing a single NCC value for three methods: Standard NCC, Lewis’ method and Luo-Konofagou method. According to Table A1, the algorithmic complexity of the FFT operation depends on the size of the tracking kernel and the corresponding search area. When the search range is small, Lewis’ method has considerably lower algorithmic complexity as compared to the standard NCC method. When the search area becomes larger, the benefit of using Lewis’ method diminishes. In contrast, the algorithmic complexity of Lou-Konofagou method is not dependent on the tracking kernel size and search range.

**Table A1.** A summary of algorithmic complexity for computing a single NCC value under the block-matching algorithm. The costs involving the construction of sum-tables is not included and analyzed below in Table A2.

		Standard NCC	Luo-Konofagou Method	Lewis’ Method
Numerator	Addition	*W*_*x*_*W*_*y*_*W*_*z*_ – 1	6	*M*_*x*_*M*_*y*_*M*_*z*_*log*_*2*_*(M*_*x*_*M*_*y*_*M*_*z*_*)/(ABC)*
	Multiplication	*W*_*x*_*W*_*y*_*W*_*z*_	None	*M*_*x*_*M*_*y*_*M*_*z*_*log*_*2*_*(M*_*x*_*M*_*y*_*M*_*z*_*)/(ABC)*
	Subtraction	None	8	None
Denominator	Addition	2(*W*_*x*_*W*_*y*_*W*_*z*_ – 1)	3	3
	Multiplication	2(*W*_*x*_*W*_*y*_*W*_*z*_) + 1	1	1
	Subtraction	None	4	4

The size of the 3-D reference signal *f* are *f*_*x*_, *f*_*y*_, and *f*_*z*_ for lateral, axial and elevation directions, respectively. The size of target signal *g* along the axial and lateral directions is the same as that of the reference signal *f*. However, the size of the 3-D target signal *g* for elevation direction is *g*_*z*_, which could differ from *f*_*z*_. The required number of operations in terms of setting up the sum tables is shown in Table A2. Recall that the standard NCC method has no need for using the sum-tables.

**Table A2.** A Summary of Algorithmic Complexity for Setting up Sum-tables needed for Lewis’ and Luo-Konofagou methods.

		Lewis’ Method	Luo-Konofagou Method
Numerator	Addition	None	3 *f*_*x*_ *f*_*y*_ ( *f*_*z*_ + *g*_*z*_ ) *− f*_*y*_ ( *f*_*z*_ + *g*_*z*_ ) *− f*_*x*_ *f*_*y*_
	Multiplication	None	*f*_*x*_ *f*_*y*_ ( *f*_*z*_ + *g*_*z*_ )
	Subtraction	None	None
Denominator	Addition	*S*_*x*_ *S*_*y*_ *S*_*z*_ (3 *f*_*x*_ *f*_*y*_ *f*_*z*_ *− f*_*y*_ *f*_*z*_ *− f*_*x*_ *f*_*z*_ *− f*_*x*_ *f*_*y*_ )	*S*_*x*_ *S*_*y*_ *S*_*z*_ (3 *f*_*x*_ *f*_*y*_ *f*_*z*_ *− f*_*y*_ *f*_*z*_ *− f*_*x*_ *f*_*z*_ *− f*_*x*_ *f*_*y*_ )
	Multiplication	*S*_*x*_ *S*_*y*_ *S*_*z*_ *f*_*x*_ *f*_*y*_ *f*_*z*_	*S*_*x*_ *S*_*y*_ *S*_*z*_ *f*_*x*_ *f*_*y*_ *f*_*z*_
	Subtraction	None	None

## Appendix C. Analysis of Memory Requirements for Sum-Table Methods

Let the size of raw RF volume data is 1024 (axial) × 128 (lateral) × 50 (elevational). The memory usage for setting up sum-table is mainly associated with 3-D search range and the size of evelational of 3-D tracking kernel. Assuming that a 3-D tracking kernel is (69 (axial) × 9 (lateral) × 3 (elevational) and the search range is 18 (axial) × 5 (lateral) × 3 (elevational), we need to access to both the reference RF volume data *f* (1024 × 128 × 3) and the target RF data *g* (1024 × 128 × 5). According to Equations (4)–(6), the RF volume data required to set up the sum-tables of *f* and *g* are 1024 × 128 × (3 + 1) and 1024 × 128 × (5 + 1). Therefore, the memory consumption for the corresponding sum-table of *f* and *g* are 1024 × 128 × (3 + 1) *× sizeo f* ( *f loat*) = 2 MB, and 1024 × 128 × (5 + 1) *× sizeo f* ( *f loat*) = 3 MB, respectively.

Since Luo-Konofagou method needs to set up a set of sum-tables at different shift locations in target signal *g* for calculation of numerator, the memory required for this part is (18 × 5 × 3) × (1024 × 128 × 4) *× sizeo f* ( *f loat*) = 540 MB. Table A3 lists the memory usage for Luo-Konofagou method and Lewis’s method given different search ranges.

**Table A3.** A Summary of memory usage for Setting up Sum-tables needed for Lewis’ and Luo-Konofagou methods.

Search Range (Axial × Lateral × Elevational)	Memory Use (MB)	
	Lewis Method	Luo-Konofagou Method
10 × 5 × 3	5	305
15 × 5 × 3	5	455
20 × 5 × 3	5	605
25 × 5 × 3	5	755
10 × 7 × 3	5	425
15 × 7 × 3	5	635
20 × 7 × 3	5	845
25 × 7 × 3	5	1055
